# A novel kernel based approach to arbitrary length symbolic data with application to type 2 diabetes risk

**DOI:** 10.1038/s41598-022-08757-1

**Published:** 2022-03-23

**Authors:** Nnanyelugo Nwegbu, Santosh Tirunagari, David Windridge

**Affiliations:** 1 Department of Computer Science, School of Science and Technology, Middlesex University, London, NW4 4BT UK; 2 Centre for Vision Speech and Signal Processing Alan Turing Building (BB), University of Surrey, Guildford, Surrey GU2 7XH UK

**Keywords:** Health care, Mathematics and computing, Computer science

## Abstract

Predictive modeling of clinical data is fraught with challenges arising from the manner in which events are recorded. Patients typically fall ill at irregular intervals and experience dissimilar intervention trajectories. This results in irregularly sampled and uneven length data which poses a problem for standard multivariate tools. The alternative of feature extraction into equal-length vectors via methods like Bag-of-Words (BoW) potentially discards useful information. We propose an approach based on a kernel framework in which data is maintained in its native form: discrete sequences of symbols. Kernel functions derived from the edit distance between pairs of sequences may then be utilized in conjunction with support vector machines to classify the data. Our method is evaluated in the context of the prediction task of determining patients likely to develop type 2 diabetes following an earlier episode of elevated blood pressure of 130/80 mmHg. Kernels combined via multi kernel learning achieved an F1-score of **0.96**, outperforming classification with SVM **0.63**, logistic regression **0.63**, Long Short Term Memory **0.61** and Multi-Layer Perceptron **0.54** applied to a BoW representation of the data. We achieved an F1-score of **0.97** on MKL on external dataset. The proposed approach is consequently able to overcome limitations associated with feature-based classification in the context of clinical data.

## Introduction

The application of supervised machine learning techniques to the medical domain has had significant impact in recent years, with clinical tasks in the areas of disease diagnosis, prognosis, and treatment all experiencing notable benefits, for example: in predicting onset of disease^1–3^, in identifying drug-to-drug interactions^4^, in phenotype discovery^5^, in risk factor identification^6^ and predictor variables^7^, predicting hospitalization^8^, suggesting suitable drugs^9,10^, prediction of type 2 diabetes mellitus (T2DM) complications^11^, in detecting adverse medical events (AMEs)^12,13^, and in developing personalized care and treatment plans^14,15^.

Extracting actionable insights has become a crucial aspect of the secondary use of clinical data, especially when integrated into a primary healthcare delivery system in the context of routine care. The success of such systems relies heavily on the effective utilization of electronic health records (EHR)^16^ (for instance, when applied in developing risk identification tools that can inform the likelihood of healthy patients succumbing to a disease). Consequently, it has become possible to leverage supervised machine learning prognosis modeling to effectively manage chronic diseases such as type 2 diabetes mellitus. In particular, it has been observed that the use of EHR in this manner to predict the onset of the disease can improve the quality and efficiency of the medical care given^1^. However, doing so requires that we overcome the challenges of predictive modeling with EHR data. This work proposes to apply a machine learning kernel framework in prognosis modeling of the likelihood of developing type 2 diabetes. It will specifically seek to address the problems of irregularly-sampled heterogeneous EHR data that are customarily found in this domain, in common with other chronic diseases requiring occasional modification and intervention to ongoing clinical treatment plans.

Type 2 diabetes is a metabolic disorder associated with patient behavior. Lifestyle intervention such as healthy diet, weight loss, and regular exercise are usually advised on diagnosis. When these fail, medication with a single non-insulin oral hypoglycemic agent is typically then prescribed. Complications such as hypertension, stroke and heart disease may occur if the target glycaemic levels are not achieved. Studies have reported that current methods of treating the disease are both uncertain and costly, and so prevention becomes an important step towards reducing the burden of care^1,17^. Thus, it has become a clinical imperative to explore predictive models based on EHR data capable of identifying those most susceptible to developing the disease, given that evidence of impending lifestyle choices can be gleaned from various clinical entities holding historic details about the patients. For instance, elevated blood pressure (BP) measurements constitute one of the key modifiable risk factors seen in people at high risk of diabetes^18^ and may help to inform intervention via early education on lifestyle choices.

Prognosis tools that carry out risk assessment such as QDiabetes^19^, FINnish Diabetes RIsk SCore (FINDRISC)^20^, and the ‘Know Your Risk’ tool from Diabetes UK^21^ are currently available online. FINDRISC is commonly used in Europe^22^. Although these tools are accessible to patients and present measures for indicating likelihood of the disease, they are unlikely to catch all susceptible patients being based on limited data (it has been found empirically that several conditions associated with increased risk of diabetes are not fully captured by Qdiabetes^23^). Thus, while these simpler models are easier to implement, they may oversimplify complex relationships that include large numbers of risk factors with nonlinear interactions^24^. In this context, UK NHS Nice guidelines on preventing type 2 diabetes recommends, where possible, computer-based risk-assessment tools using available routine EHR^25^. This is backed by evidence from studies^26,27^ indicating machine learning prognostic models developed from EHR data usually perform better than simple statistical prognostic models.

Several works have applied machine learning algorithms in identifying people at risk of developing type 2 diabetes. Recent examples include the ensemble-based approach of^1,2^, the Multi-Layer Perceptron (MLP), AdaBoost (AD), Trees Random Forest (TRF), Hidden Markov Model (HMM) of^28^, Support Vector Machine (SVM), and the Gradient Tree Boosting (GTB) approach of^29^. A previous review paper^30^ however highlighted a widespread problem of poor methodologies in developed risk tools and also the issue of inconstant use of data and predictor variables (for instance, 12 predictor variables are used in^31^ compared to 1312 predictor variables in^1^). The UK National Screening Committee report^32^, however, indicates that while a small set of risk indicators can have its advantages since they may easily be extracted from EHR data, they are less likely to include valuable information such as waist measurement and a linked family history of the disease that are strong indicators for determining the level of risk of certain patients.

Modeling with EHR is challenging despite encouraging solutions seen in several studies; the non uniform occurrence of clinical problems across populations typically results in a complex database with incomplete, sparse, and noisy data. Clinical encounters are recorded at irregularly-timed intervals and vary greatly in length and content^33,34^. Each patient record consists of a time-stamped sequence made up of a mixture of diagnosis, procedures, conditions, medications, measurements, real valued test results, administrative, demographic and other relevant information. A patient profile must thus be derived from a mixture of these diverse entities. Patient representation with time-dependent interactions^35^ makes it difficult to apply traditional statistical pattern recognition tools. As a result, simpler representations such as aggregate features (eg, event count and event average)^36^ are often sought.

A combination of techniques can be applied to deal with these data issues. The usual model of bespoke feature selection is time consuming and the treatment of sequential data via feature vectorization is typically both information-losing and inappropriately constrained to an arbitrarily-fixed dimensionality. It can also lead to a selection bias. Imputation is one of the methods used to overcome issues with missing values. For example, mean or median value imputation^23^ for numeric data or Random Forest^11,27^ for both categorical and numeric data. Interpolation with Gaussian process regression (GPR)^37^ also referred to as kriging is adopted to address the problem of irregularly sampled data. Deep learning methods such as RNNs and LSTM^36,38^, while inherently tuned towards regular data sampling, can be adapted to irregularly sampled and uneven sequential data. Variations in length and interval between entities may themselves hold informative value; especially in regard to health status and how often a patient utilizes healthcare.

Transforming raw data into features is thus, even within a deep learning context, a key step in exploiting the inherent structure of data within a given learning domain. In the health-care domain, this means seeking identical feature representations for patients with similar traits and characteristics. Consequently, the majority of studies surveyed^39^ implicitly embody the idea of *representation learning*.

By contrast, we shall seek in this paper to propose an efficient method for performing EHR modeling that retains the *intrinsic* representation of the data as a discrete sequence of symbols, thereby treating the problem as a featureless pattern recognition task, allowing us to apply machine learning algorithms to EHR data while retaining its spatial and temporal aspects (it has been argued elsewhere that solutions based on symbolic data sequences outperform those based on conventional crisp data^12,40^). We shall argue, in particular, that EHR symbolic sequence representation is amenable to the application of error-tolerant elastic dissimilarity measures such as edit distance that can deal with distortions of the unequal length sequences while at the same time retaining the essential data characteristics.

Edit distance measures how dissimilar two sequences are by counting the number of edit operations needed to make them equal. Common edit operations include, insert, delete, and substitution. It has been used in natural language processing (NLP) and time series classification tasks^12,41–44^. It is suited for distance substitution methods in algorithms that implement a distance computation between objects. It can be applied directly in metric based classification methods such as K-Nearest Neighbour (KNN) by replacing the Euclidean distance. Alternatively, it can be incorporated in a similar manner with pairwise similarity functions defined as the Gaussian kernel or as norm computation within the kernel learning framework^42–45^.

The kernel framework is a modular approach that implicitly maps (in a potentially non-linear manner) data points into an embedded high dimensional linear feature space where linear separation/classification can take place. It is computationally efficient in that we do not need to explicitly compute the coordinates of the points in the embedded space; rather we apply a pairwise similarity function (also called a kernel function) directly on the raw input data. The kernel function corresponds to an inner product between each pair of points in the embedded feature space (known as the ‘kernel trick’). A valid kernel obeys the Mercer condition and is thus guaranteed to yield a symmetric and positive semi-definite (PSD) kernel matrix. This kernel matrix serves as the input into a kernel based discriminatory learning algorithm. Optimization problems formulated in terms of the kernel are independent of the dimensionality of the raw input, for example, solving the optimization problem that finds the hyperplane that maximally separates the labeled data points in a kernelised SVM. This is a key advantage of the kernel framework and makes it well suited for high dimensional data with fewer examples.

Edit distance and its variants have been shown to be useful in designing string edit kernels for solving sequence alignment problems (spectral modification methods may be adopted to ensure the kernels are PSD; SVM solvers may also be adapted to converge to a solution even with non PSD kernels). In addition, kernels can also be combined linearly to form further kernels as in multi kernel learning (MKL)^46^. Here, a kernel weight quantifies the contribution of an individual kernel matrix to the convex combination, the collective coefficients for which are determined via the MKL optimization process. MKL thus makes it possible to combine kernels derived from disparate sources, especially heterogeneous non vectorized data types. We shall argue that this makes a kernel-based framework a suitable approach for dealing with the heterogeneous sequential data types presented by EHRs.

This study is hence focused on addressing the problem of modeling EHR data within such a kernel framework. In particular, we propose that bespoke Edit kernels may be used to address the problem of uneven length data, while MKL enables us to process heterogeneous data within a single classifier.

### Datasets

Experiments are conducted using anonymised test dummy primary health care data that reflects actual medical data. It is modeled after Vision 3 General Practice IT system data. We searched the database for patients with a read code—C10..00 for Diabetes Mellitus recorded. 158 out of 9628 patients that met the inclusion criteria were used for this study. Each patient record was checked for the presence of systolic BP of 130 mmHg and diastolic BP of 80 mmHg recorded prior to being diagnosed with type 2 diabetes. The presence or absence of elevated blood pressure was used as the outcome variable. Those identified with prior blood pressure equal to or exceeding 130/80 mmHg were labelled as positive, while those with reading less than 130/80 mmHg were labelled as negative. Of the 158 patients, 42% (66) were labelled as positive while 58% (92) were labeled as negative. The self-identified gender distribution is 76 females, 82 males.

**Figure 1:**
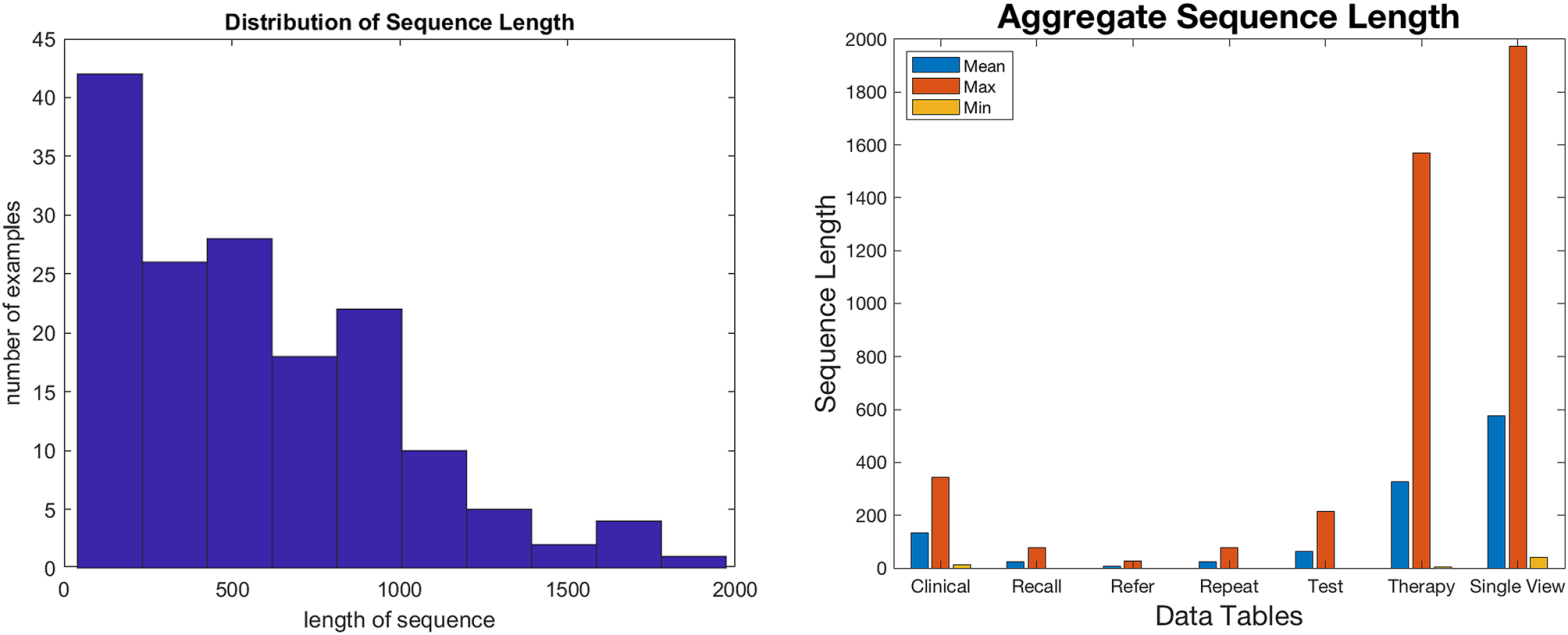
Plots showing the uneven sequence length distribution and the aggregate mean, maximum and minimum length distribution according to the datasets.

Each patient record consists of a discrete symbolic sequence of length between 40 to 1974 data items. The distribution of the variable length sequences is displayed in Fig. 1. Table 1 shows a sample of data extracted for a single patient. The read codes ordered by the event dates are extracted as the discrete symbols. The data cleansing carried out is documented in the Supplementary Data Preprocessing/Cleansing section of the Supplementary text.

**Table 1:** A sample of symbolic representation a patient data.

Event date	Read code	Read term
19951024	C10..00	Diabetes mellitus
19951024	137..00	Tobacco consumption
19951030	136..00	Alcohol consumption
19951114	229..00	O/E-height
19951114	22A..00	O/E-weight
19951202	246..00	O/E-blood pressure reading
19951202	115..00	No significant medical history
19951202	1225.11	No FH: CVA/stroke/TIA

The Read codes are extracted as a sequence of symbols and ordered according to the event date.

#### Reference validation dataset

We compare the performance of our method against publicly available UCI machine learning data—membranolytic anticancer peptides (ACPs) (Available at [https://archive.ics.uci.edu/ml/datasets/Anticancer+peptides]). The data is made up of one-letter amino-acid sequences for breast cancer and lung cancer cells. It was used in an ensemble machine learning study^47^ that identified anticancer peptides.

The dataset consists of 4 classes (inactive-exp, inactive-virtual, moderately active and very active) distributed according to 83, 750, 98 & 18 examples respectively. The length of sequences ranges between 5 (minimum) and 38 (maximum) with a mean length of 17 and 5.5 standard deviation. No data cleansing or pre-process steps were performed on the dataset.

We applied our model to identify the examples that belong to the ‘inactive-virtual’ class. Treating the task as a multi-class learning problem, we apply the one-vs-all method to select all members of the ‘inactive-exp’ (83), ‘mod active’ (98) and ‘very active’ (18) classes to make up the negative class (199) while an equal number of examples (199) was selected from the ’inactive-virtual’ class as the positive examples. We use an equal distribution of both classes to avoid introducing bias from class imbalance.

An initial partition of 60% for training, 20% for validation and 20% for testing datasets was adopted for cross validation; however to ensure a consistent approach was adopted for all experiments, we combined the test and training datasets and applied the Leave-One-Out (LOOCV) cross validation instead. In addition, 10 objects were set aside as the set of zero vector sequences. These were used in crafting kernel functions using the distance substitution approach. The leave-one-out data was distributed according to 156 positive and 152 negative examples.

## Methodology

In this section, we introduce the edit distance dissimilarity measure with variants of edit kernels derived from it. The distance substitution approach was applied in defining the kernel functions.

### Problem definition

We address the problem of developing an effective data-driven approach to predicting people at risk of developing type 2 diabetes. The patient’s behavior and characteristics were extracted from various relational databases that make up the EHR database. These heterogeneous timestamped clinical and non-clinical events exist in categorical and numerical real valued test measurements. The motivation for the selected models stems from the need to exploit heterogeneity within the data, specifically by incorporating both spatial and temporal information regarding patient behavior, at the same time overcoming the problem of irregularly sampled data of uneven length. This study seeks to address the question of how to characterize elevation in blood pressure of 130/80 mmHg in healthy patients in serving as a warning for developing type 2 diabetes. Elevated BP is a modifiable risk factor that is also monitored in people at risk of developing hypertension. A key question is therefore whether healthy patients with an occurrence of elevated BP prior to the onset of type 2 diabetes share similar behavior? If such patterns exist within the data, can the approach adopted in this study serve as a preventive measure?

In order to achieve this;

- Variants of the edit distance kernels are developed and applied to the data.
- A search for the best predictive value is carried out by applying a multi kernel learning (MKL) approach.

### Kernel definition

**Figure 2:**
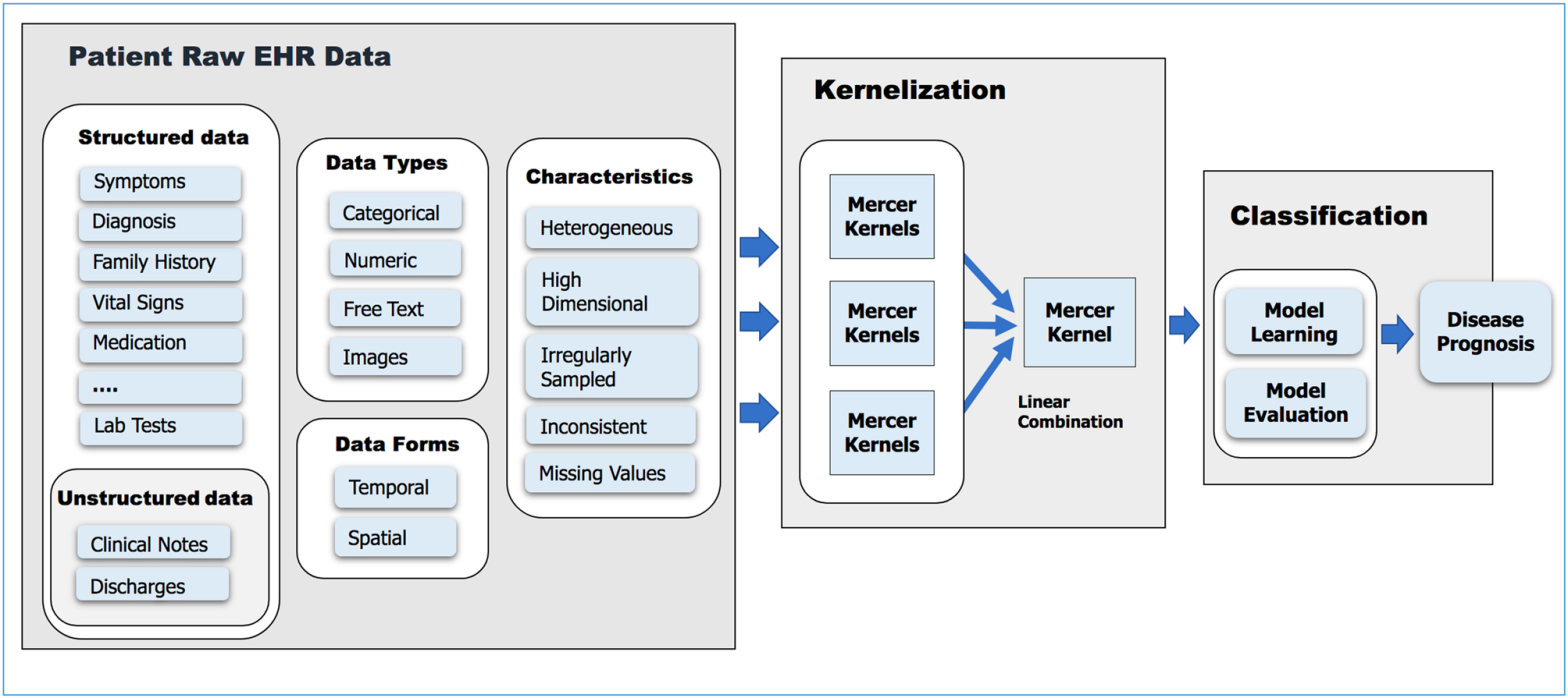
The proposed kernel framework for disease prognosis modeling with EHR data.

Via the so-called kernel trick, a kernel is equivalent to an implicit mapping of entity pairs into a high dimensional feature space followed by a vector product in that space. It is thus a symmetric function $K : X \times X \mapsto {\mathbb {R}}$ such that,

1 $$\begin{aligned} \forall \; {x_i}, {x_j} \in X, \; \; k({x_i},{x_j}) = \langle \phi (x_i), \phi (x_j) \rangle \end{aligned}$$

where $\phi : X \mapsto F$ is a function map $\phi$ that transforms the input *X* into a high dimensional feature space *F*. The notation $x_i$ used in this paper corresponds to a single patient sequence of symbols, such as those encoding clinical interventions, symptoms, diagnosis, procedures, and medication. A valid kernel function is positive definite if it satisfies the condition

2 $$\begin{aligned} k(x_i,x_j) = \sum _{i,j = 1}^{n}c_i,c_j k(x_i,x_j) \ge 0 \end{aligned}$$

for any $x,..,x_n \in X$ and $c,\ldots,c_n \in {\mathbb {R}}$ or, equivalently, that all eigenvalues of its matrix are non-negative. See Fig. 2 for the conceptual framework.

Distance measures are metrics that obey the triangular inequality. They generate non-negative values and have zeros along the diagonals of its symmetric matrix. Since PSD kernels are generalizations of vector products in the induced Mercer feature space we can extend the concept of PSD kernels to a larger class of kernels known as conditionally positive kernels (cpd) expressed in terms of norms of the embedding feature space. Thus, the norm $||\phi (x_i) - \phi (x_j) ||^2$ quantifying how close objects are in the feature space can be expressed in terms of the kernel function:

3 $$\begin{aligned} ||\phi (x_i) - \phi (x_j) ||^2 = k({x_i},{x_i}) + k({x_j},{x_j}) - 2k({x_i},{x_j}) \end{aligned}$$

where *k*(., .) is a kernel function

As a result, we are able to apply distance metrics, in this case edit distance, in the construction of kernels. A distance measure is said to be isometric to the L2-norm if the data can be embedded in a Hilbert space such that $d(x, x_0) = ||\phi (x) - \phi (x_0)||$ (this approach is termed a ‘distance substitution kernel’).

This intuition stems from norms being invariant to translations, $x \mapsto x_i-x_0$ in contrast to dot products. The dot product of the translation can be expressed as

4 $$\begin{aligned} \langle (x_i- x_0) , (x_j-x_0) \rangle = \frac{1}{2}(-||x_i - x_j||)^2 + ||x_i - x_0||)^2 + ||x_0 - x_j||)^2 \end{aligned}$$

For any $x_0 \in X$ we show this to be a valid PSD kernel by

5 $$\begin{aligned} \sum _{i,j }c_i,c_j\langle (x_i- x_0),(x_j-x_0) \rangle = \sum _{i,j }c_i|| (x_i - x_0) ||^2 \ge 0 \end{aligned}$$

A conditionally positive definite symmetric $n \times n$ matrix K $(m \ge 2)$, on the other hand, also satisfies the condition in Eq. (2) for any $x,\ldots,x_n \in X$ and $c,\ldots,c_n \in {\mathbb {R}}$ but with the additional property $\sum _{i= 1}^{n}c_i = 0$

For kernelised SVMs, positive semi-definiteness guarantees convexity of the problem and thereby convergence; non PSD kernels may also converge in practice, but are not guaranteed to do so. Nevertheless, it is still possible to learn directly from non-PSD kernels and obtain good classification results in practice, though solutions from non-PSD kernels may be hard to interpret due to a missing geometrical and theoretical intuition^48^. Spectral modifications^49^ can also be applied to the negative eigenvalues in order to make the matrix PSD, for instance by clipping to remove the negative eigenvalues, shifting the entire spectrum till the least eigenvalue is 0, flipping to use the absolute value of the spectrum, or squaring of the kernel matrix.

### Edit distance

Edit distance has been found to be general and accurate measure of sequence dissimilarities^50^. It requires computing the minimum number of edit operations needed to convert one sequence into another. Commonly used edit operations are insert, delete and substitution. A non negative value is assigned to each edit operation and the minimum total cost in transforming one sequence into another is selected. The process involves traversing both sequences one symbol at a time and assigning a zero cost if both symbols match. If however they differ, we recursively compute the cost of all edit operations and select whichever has the least cost.

Given discrete sequences, $x = x_1,\ldots,x_i$ and $y = y_1, \ldots ,y_j$ derived from Alphabet $\Sigma$, the edit distance between the two sequences $k_{ed1}(x,y)$ is computed recursively via dynamic programming applied to the following equation^51^

6 $$\begin{aligned} d_{ij} = min \left\{ \begin{array}{ll} d_{i-1,j} + 1 \\ d_{i,j-1} + 1 \\ d_{i-1,j-1} + (\text {if} \; x_i\, = y_j\; \text {then}\; 0\; \text {else} \; 2) \end{array} \right. \end{aligned}$$

Where the distance table ($d_{i,j}$) tabulates the edit distances $k_{ed1}(x_1 \ldots x_i, y_1\ldots y_j)$

### Edit kernel construction

- *Edit kernel*. Here we construct kernel functions with the distance substitution method by replacing the norms in Eq. (4) with the edit distance. By treating the data as a sequence of symbols, we can use the elastic edit distance as a dissimilarity measure to overcome the problem of uneven length sequences. We compute the edit distance from pattern *x* to ${x_0}$ and from ${x_0}$ to ${x'}$ in relation to the distance from *x* to ${x'}$, as detailed in Eq. (4).
  7 $$\begin{aligned} K_{1}(x,x') = \frac{1}{2}(d{(x,x_0)}^{2} + d{(x_0,x')}^{2} - d{(x,x')}^{2}) \end{aligned}$$
  where $d(.,.)^2$ is substituted by the edit distance $k_{ed1}(x,x_0)$ of two symbols. We can therefore re-write Eq. (7).
  8 $$\begin{aligned} K_{1}(x,x') = \frac{1}{2}(k_{ed1}{(x,x_0)} + k_{ed1}{(x_0,x')} - k_{ed1}{(x,x')}) \end{aligned}$$
  We can construct additional kernel functions by defining variations of the edit distance computation.
- *Edit kernel with length normalization*. For this kernel function, we apply a normalizing factor *N* to the edit distance computation. Normalizing with the length of the longer sequence takes into consideration any effect the length of the sequences may have on the proximity of the pair of data points.
  9 $$\begin{aligned} k_{ed2}(x_i,x'_j) = \frac{ k_{ed1}(x_i,x'_j)}{N} \end{aligned}$$
  where *N* is the length of the longer sequence.
- *Edit kernel normalized by number of common items*. We create this kernel function by normalizing the edit distance computation by the number $|x_i \cap x'_j|$ of common elements between both sequences.
  10 $$\begin{aligned} k_{ed3}(x_i,x'_j) = \frac{ k_{ed1}(x_i,x'_j)}{|x_i \cap x'_j|} \end{aligned}$$
- *Edit kernel normalized by exponent of number of common items*. The normalization factor used in the construction of this kernel is scaled exponentially to $\lambda$ = $2^{|x_i \cap x'_j|}$
  11 $$\begin{aligned} k_{ed4}(x_i,x'_j) = \frac{ k_{ed1}(x_i,x'_j)}{\lambda } \end{aligned}$$
  The indicated variations of the edit distance computation $K_{ed2}$, $K_{ed3}$ and $K_{ed4}$ are used in the same distance substitution manner in Eq. (8) to construct additional kernel functions $K_2$, $K_3$, and $K_4$

### Learning algorithms

**Figure 3:**
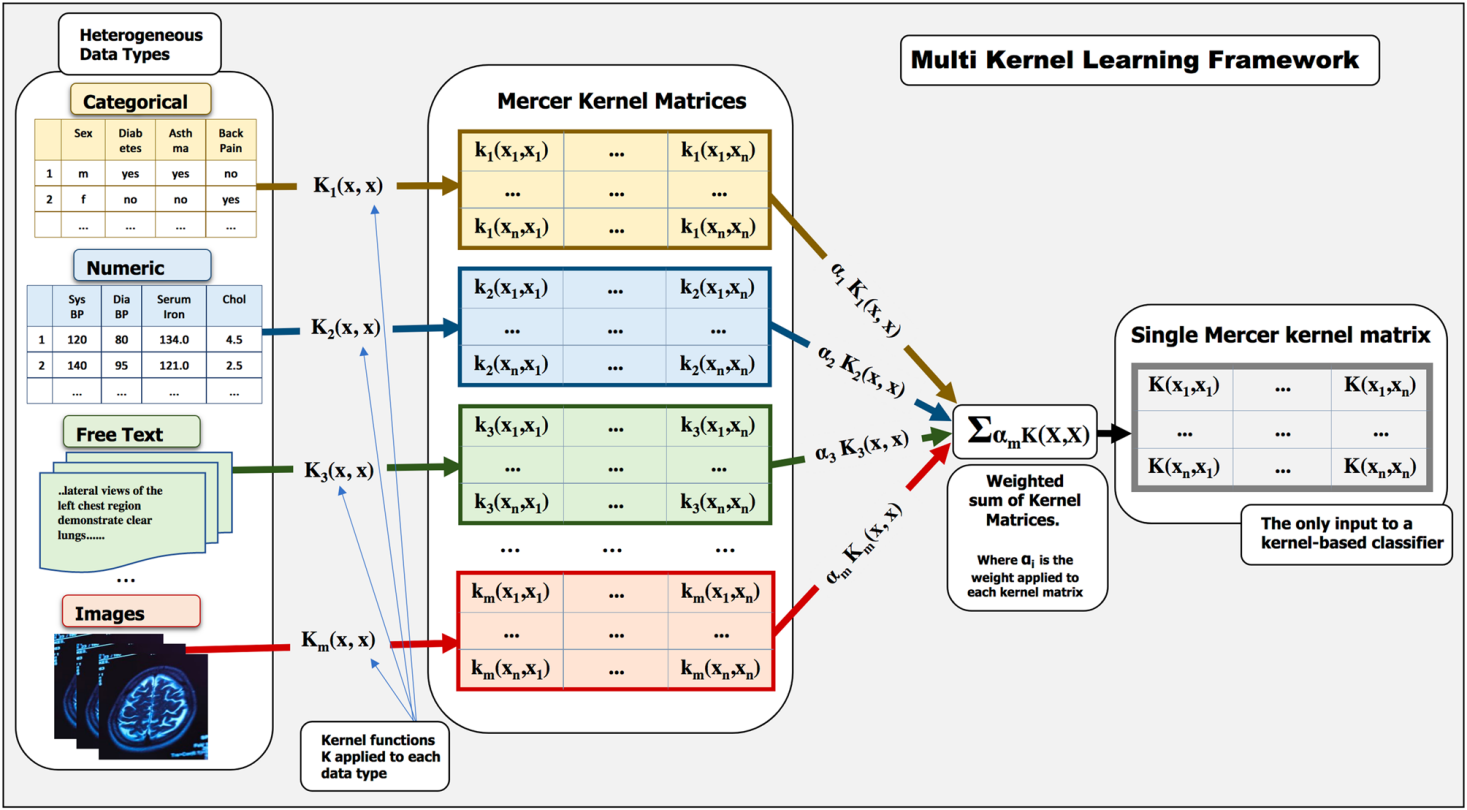
The multi kernel learning (MKL) framework for combining kernels derived from disparate data types.

We train our dataset using a SVM classifier implemented with LibSVM^52^ algorithm (SVM is the commonest of the kernel based machine learning classifiers that can discriminate complex datasets, seeking the maximum separating hyperplane between two classes of data). A convex sum of kernels is also a valid kernel. It is therefore possible to convexly combine multiple kernels and optimize over the weight coefficients in the classification learning process. We use SimpleMKL^46^ to achieve this in the MKL experiments (see Fig. 3 for the MKL framework). More details about these algorithms are provided in the supplementary file. We use kernel alignment and classification performance as a means to evaluate the suitability of kernel functions. The F1-score, accuracy, sensitivity, specificity and number of support vectors are computed and used as performance metrics.

### Classification performance and evaluation

Given the number of examples, Leave One Out Cross Validation (LOOCV) was used to evaluate the classification performance. Each data point was used as the test example while the rest served as the training examples.

The mean of the following performance metrics, true positive TP, true negative TN, false negative FN and, false positive FP of the predicted labels was calculated. The classification accuracy is given as the ratio of the correct positive and negative predicted outcomes against all predicted outcomes.

$$\begin{aligned} Accuracy = \frac{TP+TN}{TP+FP+FN+TN} \end{aligned}$$

This however does not give the true reflection of performance if there is a class imbalance from an uneven distribution of positives and negative outcomes. In addition, precision which measures the ratio of the correctly predicted positive labels to the total number of all predicted positive labels $\frac{TP}{TP+FP}$ and the recall (sensitivity) that measures the ratio of the correctly predicted positive labels to all the actual positive labels $\frac{TP}{TP+FN}$ were also derived. A single metric, the F1-score, combines these two and is derived by calculating the harmonic average of the precision and recall.

$$\begin{aligned} \text{F}1{\text{-score}} = 2* \frac{Recall * Precision}{Recall + Precision} \end{aligned}$$

This gives better performance evaluation where class imbalance exists since it takes into account the wrong predicted positive and negative labels.

There is no other clinical basis for which to separate the cohort into a test/train set (i.e no experimental control group).

### Baseline models

**Figure 4:**
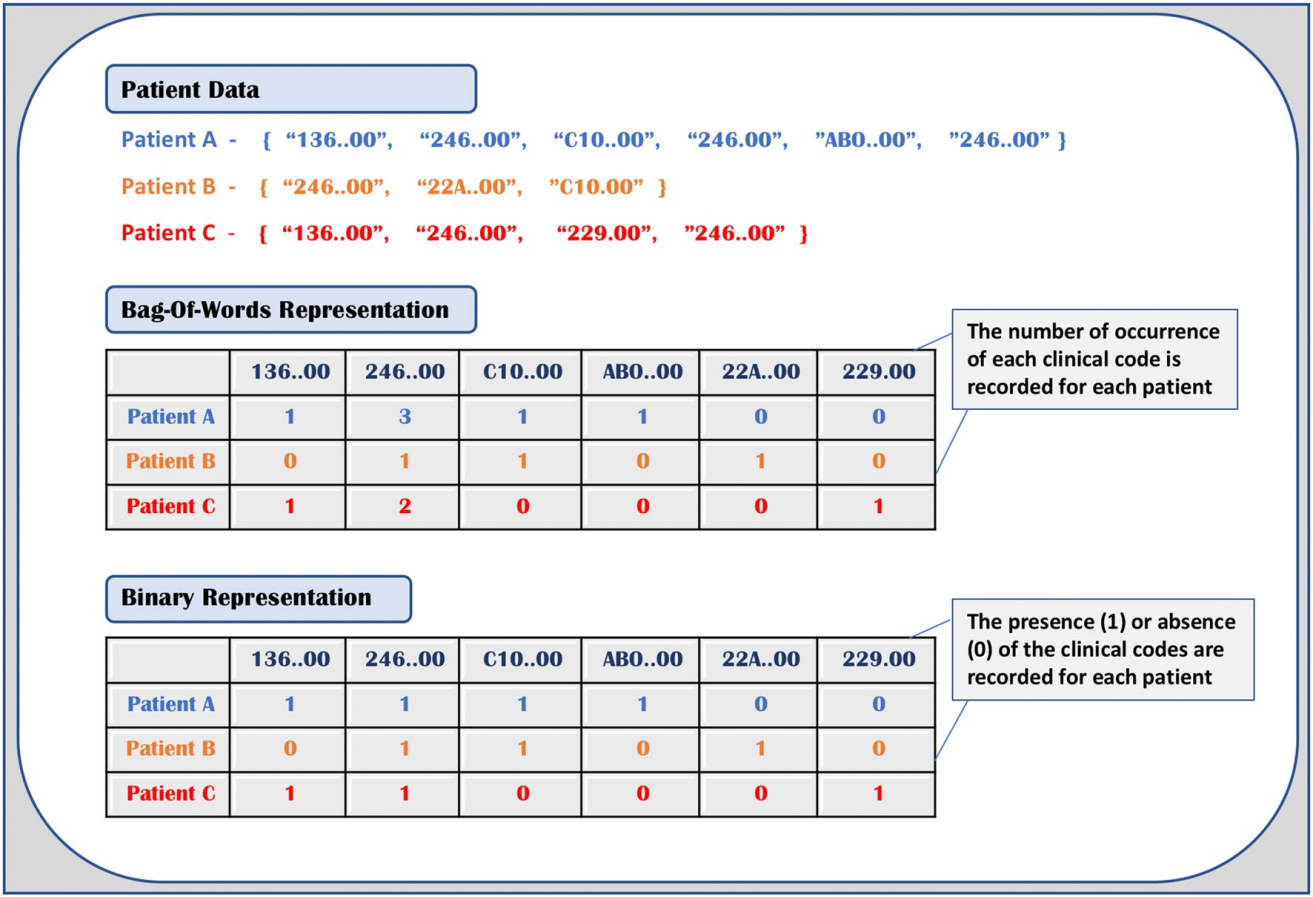
An illustration of the feature extraction process into Bag-of-Words and Binary features for 3 sample patient data.

We compared the proposed framework against established classical methods used in feature representation and classification of sequential data, such as in natural language processing (NLP) tasks. We adopt the Bag-of-Words (BoW) method of extracting even-length numeric feature vectors. Bag-of-Words yields a histogram of data entities representing the frequency of occurrence for each patient. The tabular data matrix contains 3054 unique clinical codes with each code per column. The extraction method is illustrated in Fig. 4. In contrast, the binary feature representation encodes the presence or absence of the clinical codes for each patient record.

The following classification algorithms, logistic regression, SVM, and Deep learning recurrent recurrent neural network (RNN) with long short-term memory (LSTM) are used to classify the data. We use these as the baseline to evaluate the classification performance and suitability of our proposed method.

#### Baseline kernel functions

The following base kernel functions were applied with SVM as the baseline models

- *Linear* The linear kernel is the dot product similarity measure and one of the basic kernels used with SVM for linearly separable data.
  12 $$\begin{aligned} K(x,y) = (x^{\mathsf {T}}y ) \end{aligned}$$
- *Polynomial kernel* The polynomial kernel is defined as
  13 $$\begin{aligned} {\displaystyle K(x,y)=(x^{\mathsf {T}}y+c)^{d}} \end{aligned}$$
  where *d* is the degree of the polynomial and $c \ge 0$ is a free parameter that controls the influence of higher order terms of the polynomial.
- *Radial basis function (RBF) kernel* The Radial Basis Function (RBF) kernel, also referred to as the Gaussian kernel, is well suited for numeric data. It has some interesting properties that makes it suitable for a lot of classification tasks. Its free parameter $\frac{1}{2\sigma }$ can be used to control the performance of the kernel
  14 $$\begin{aligned} {\displaystyle K(x,y)=exp\left( - \frac{\big |\big |x - y \big |\big |^{2}}{2\sigma } \right) } \end{aligned}$$
- *Exponential RBF kernel* The exponential RBF kernel differs from the Gaussian RBF kernel by its norm which is not squared.
  15 $$\begin{aligned} {\displaystyle K(x,y)=exp\left( - \frac{\big |\big | x - y \big |\big |}{2\sigma } \right) } \end{aligned}$$
- *Laplace kernel* This kernel function is also a part of the RBF family of kernels. It is similar to the exponential RBF only that it is not too sensitive to its free parameter $\sigma$
  16 $$\begin{aligned} {\displaystyle K(x,y)=exp\left( - \frac{\big |\big | x - y \big |\big | }{ \sigma } \right) } \end{aligned}$$

## Experimental objectives

Our experimental procedures and objectives are as follows:

- *Effect of different kernel functions* The suitability of bespoke kernel functions derived from an edit distance measure between a pair of EHR sequences is investigated. The four variants described in the methodology section are applied to the data. Our working hypothesis is that edit distance measures in general address the problem of irregularly-sampled uneven length data, with experiments used to establish how each of the kernel function variants affects classification performance.
- *Single vs multiple kernels* A single kernel matrix expresses the data distribution and structure within it’s corresponding induced feature space. Each kernel function therefore denotes a different expression of, or window on, the underlying sequential pattern structure. A weighted combination of multiple kernels with MKL allows us to forgo the problem of determining which is the most discriminative of the edit-distance kernel variants. We can also use this approach to combining kernels to integrate data derived from heterogeneous longitudinal data sources. We will thus investigate and compare the classification performance of single kernels against multiple kernels.
- *Comparison with traditional Bag-of-Words (BoW)* To demonstrate the effectiveness of our method, we compare the suggested kernel framework against the conventional models based on BoW features. This serves as the baseline to evaluate the predictive performance of our model as a suitable disease prognosis tool.
- *Validation on external data* We further validate the suitability of the proposed model via an experiment designed to test the robustness of our model as a solution to predictive modeling of uneven-length sequences of symbolic data.

The kernel function evaluation applied to sequence-pairs computes a real valued quantity that signifies how similar the objects are.

**Figure 5:**
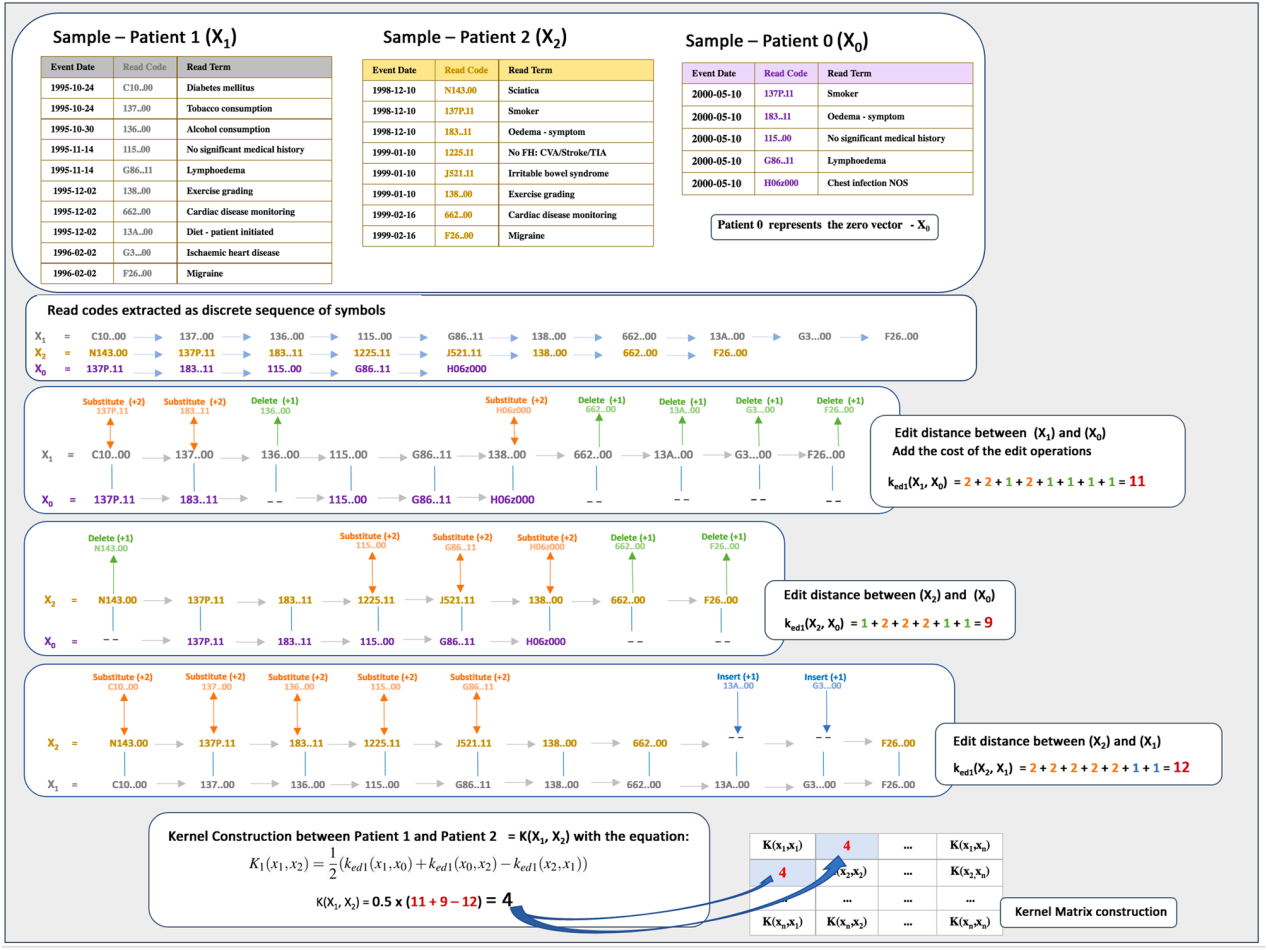
Evaluating the pairwise kernel function by first extracting the data as a sequence of symbols, then computing the edit distance between a pair of sequences. An edit cost of 2 is applied where symbols are *substituted* while 1 is applied if a symbol is *deleted* or *inserted*. The total cost is computed and used to derive the kernel function value as specified in Eq. (8).

This experiment applies the distance substitution method in constructing kernel functions. Codes ordered by the event dates are first extracted for each patient (see Table 1 for a sample data for a patient). In order to evaluate a kernel function between a pair of sequences, we compute the translation to the origin for each sequence in reference to the edit distance between the two sequences (see Eq. (8) for the function definition and Fig. 5 for an illustration of the process). By translation to the origin we mean subtraction of the edit distance between each sequence and the determined zero vector, where the zero vector is a candidate patient selected from the dataset.

The edit distance between two sequences requires that we traverse each pair of symbols and compute the minimum cost of converting one symbol into the other. We may either insert or delete a symbol at unit cost or we substitute the symbols at an edit cost of 2.

## Results

### Effect of different kernel functions

**Figure 6:**
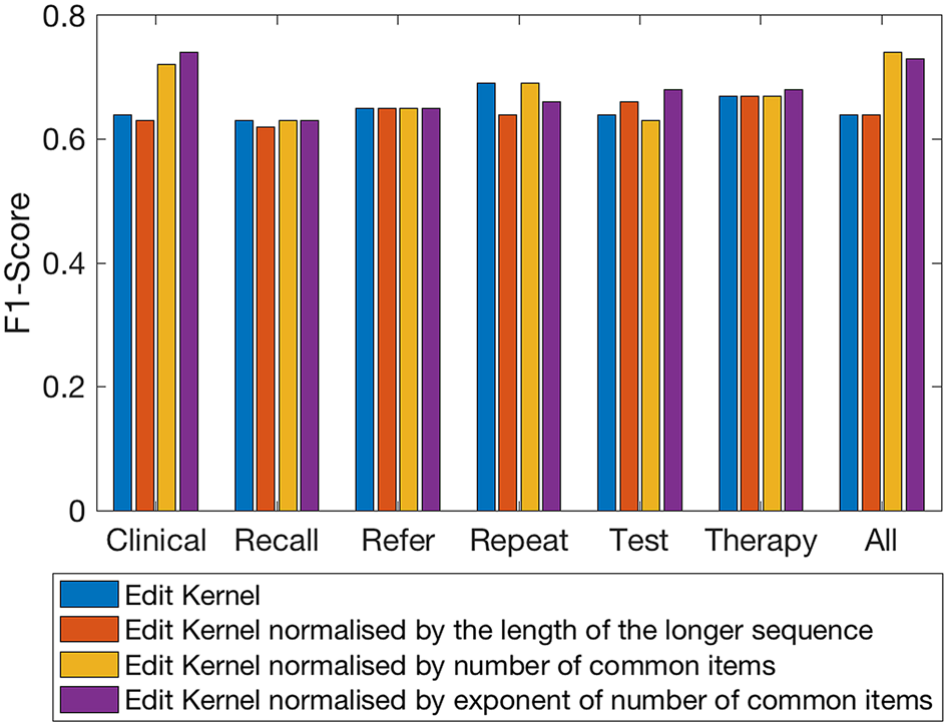
Bar chart showing the F1-scores obtained by applying the 4 kernel functions to the datasets.

**Figure 7:**
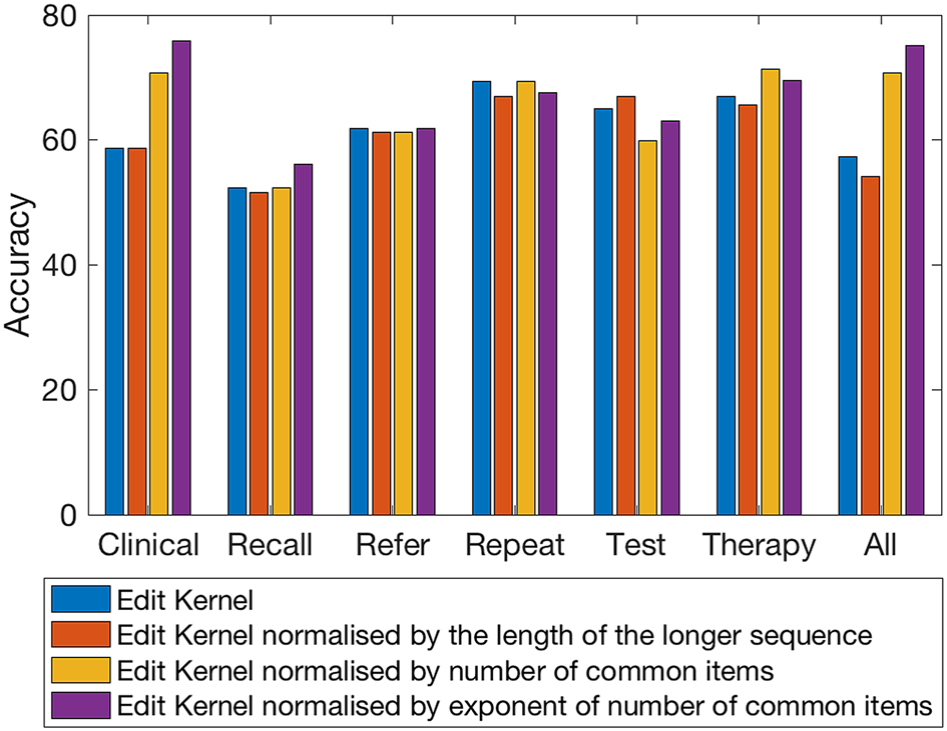
Bar chart showing the corresponding Accuracy obtained by applying the 4 kernel functions to the datasets.

In order to verify the suitability of the kernel framework, we evaluate the discriminative performance of the 4 edit kernel functions executed on the uneven length symbolic sequences extracted from the 6 data tables (i.e. data modalities). Firstly, we experiment on data extracted from the distinct relational data tables they were originally stored in and secondly as a collective single view data for each patient. In both cases, the symbolic sequences are ordered by the event dates. We employ a greedy search to obtain the most suitable zero vector by running the classification process 158 times. Each patient sequence is excluded from the dataset and used as the zero vector sequence in turn (see Figs. 6 and 7 for an illustration of the F1-score and Accuracy obtained).

We further augment the determination of kernel discriminative power by applying kernel post processing to generate 2 additional variants of each kernel matrix. First, we run the classifier on the kernel matrix in its original raw form, secondly, the kernel is normalised and lastly the normalised kernel is centralized. By centralizing the kernel matrices, we assess in isolation the usefulness of translation by the zero vector to the origin of the feature space. Using Leave-One-Out cross validation, we apply the LibSVM algorithm in a heuristic manner with 4 regularization parameters C to classify the data.

The greatest F1-score of 0.74 (see Table 2) was achieved via the ‘Edit Kernel normalised by exponent of number of common items’ kernel applied to the clinical dataset with the same score also achieved with the ‘Edit Kernel normalised by the number of common items’ kernel applied to the full complement dataset. In both cases, the post-normalized kernel matrices produced the best result.

**Table 2:** Best results obtained from classification with single kernels constructed from the datasets, where X_0_ : zero vector index; F1: F1-score; Acc: accuracy; Sen: sensitivity; Spec: specificity; nSV: number of support vectors.

Tables	X_0_	F1	Acc (%) ± (std)	Sen	Spec	nSV
Clinical	121	0.74	75.80 (42.97)	0.80	0.73	18
Recall	61	0.63	56.05 (49.79)	0.95	0.21	13
Refer	48	0.65	61.78 (48.75)	0.85	0.45	8
Repeat	55	0.69	69.43 (46.22)	0.82	0.60	90
Test	20	0.68	63.06 (50.04)	0.95	0.40	102
Therapy	4	0.67	66.88 (47.22)	0.80	0.58	55
All data	41	0.74	70.70 (45.66)	0.98	0.51	18

### Single vs multiple kernels

**Figure 8:**
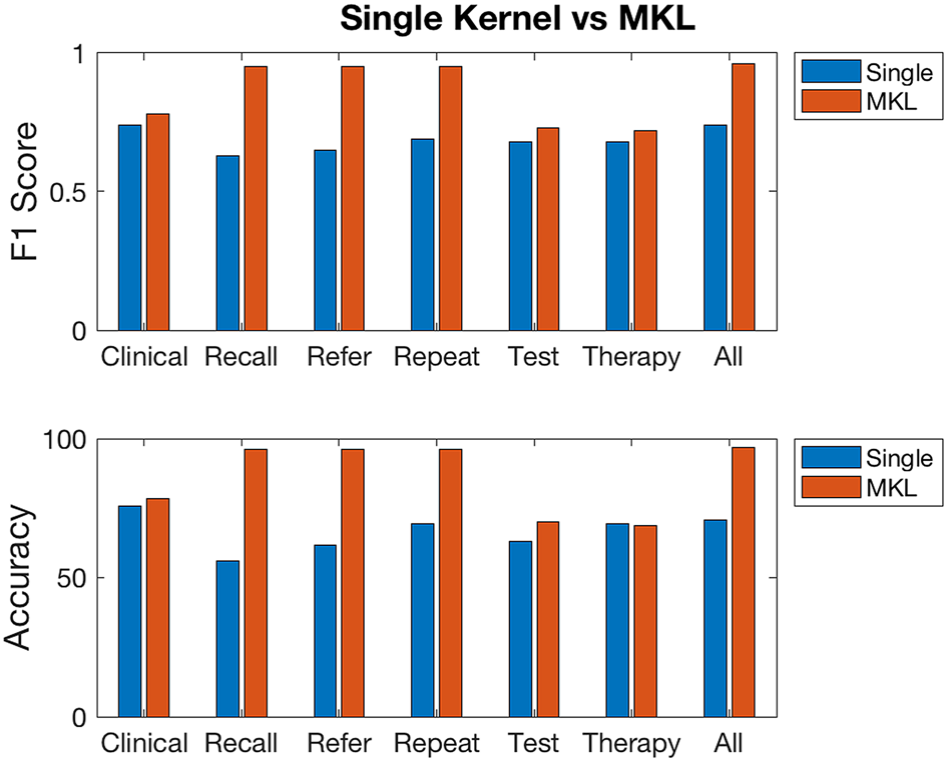
Best F1-score and accuracy achieved from classification with a single kernel vs MKL.

Combining kernels algebraically into a single model potentially offers the possibility of an enhanced representation of the patterns we are seeking to exploit. Using SimpleMKL, we seek the optimum linear combination of the four kernel functions for each dataset. This allows us to evaluate and compare the performance achieved via single kernel learning vs MKL. MKL results achieved for each dataset are shown in Table 3. An F1-score of 0.96 was obtained with the single view dataset while 0.95 F1-score was obtained with the Recall, Refer, and Repeat datasets. The plots in Fig. 8 show the comparison between the F1-score and Accuracy of the single kernel vs MKL performance. We also show the learned MKL combination weight coefficients $\sigma$ in Table 1 of the supplementary file. The entire 24 kernel matrices resulting from 4 kernel functions applied to each data set was also combined via MKL into a single classification model. This model achieved an F1-score of 0.92 as may be seen in Table 4.

**Table 3:** Best results obtained from MKL convex optimization combining the four kernels applied independently to the respective data tables.

Tables	X_0_	F1	Acc (%)	Sen	Spec	nSV
Clinical	88	0.78	78.34	0.89	0.70	112
Recall	44	0.95	96.18	0.91	1.00	124
Refer	97	0.95	96.18	0.91	1.00	127
Repeat	85	0.95	96.18	0.91	1.00	127
Test	110	0.73	70.06	0.97	0.51	75
Therapy	3	0.72	68.79	0.97	0.49	105
All data	37	0.96	96.82	0.95	0.98	116

**Table 4:** MKL results obtained from combining 24 kernel matrices derived from the datasets (4 kernels per dataset).

Tables	X_0_	F1	Acc	Sen	Spec	nSV
All kernels	90	0.92	94.27	0.86	1	41

### Comparison with traditional Bag-of-Words (BoW)

BoW feature representation is one of the traditional methods commonly used to represent sequential data in vectorial form. We apply logistic regression, SVM and deep learning Multi-Layer Perceptron (MLP) and long short term memory (LSTM) to the BoW features as a baseline to compare our model against. Table 5 and 6 shows the results obtained. The SVM is tested with the standard kernel functions, linear, polynomial, RBF, Exponential RBF and Laplace which work well with even-length vectorised data. Experimenting with the same leave-one-out cross-validation (LOOCV), the F1-score of **0.63** and accuracy of **69.62** was achieved with SVM implementing the Laplace kernel on the Bag-of-Words features. The deep learning models with results displayed on Table 6 were also partitioned using the LOOCV approach. The LSTM on the Binary Bag-of-Words outperformed the LSTM on regular Bag-of-Words features with an F1-score of **0.61** and accuracy of **51.27**.

**Table 5:** Performance result obtained with logistic regression and SVM applied to Bag-of-Words and Binary Bag-of-Words feature representation of the data.

Models		Bag-of-Words				Binary Bag-of-Words			
		F1	Acc (%) ± (std)	Sen	Spec	F1	Acc (%) ± (std)	Sen	Spec
SVM	Linear	0.51	56.96 (50.00)	0.49	0.64	0.61	67.09 (47.00)	0.61	0.72
	Poly	0.62	62.03 (49.00)	0.53	0.74	0.59	67.09 (47.00)	0.61	0.71
	RBF	0.25	62.03 (49.00)	0.71	0.61	0.25	62.03 (49.00)	0.61	0.71
	Exp RBF	0.20	60.76 (49.00)	0.67	0.60	0.18	57.59 (49.00)	0.47	0.59
	Laplace	0.63	69.62 (46.00)	0.65	0.73	0.62	68.99 (46.00)	0.63	0.73
Logistic regression		0.51	56.96 (50.00)	0.49	0.64	0.63	70.25 (45.00)	0.65	0.74

**Table 6:** Performance result obtained with deep learning LSTM and MLP applied to Bag-of-Words and Binary Bag-of-Words feature representation of the data.

Models		Bag-of-Words				Binary Bag-of-Words			
		F1	Acc (%)	Sen	Spec	F1	Acc (%)	Sen	Spec
Deep learning	MLP	0.54	60.76 (48.98)	0.53	0.67	0.46	59.49 (49.25)	0.52	0.63
	LSTM	0.44	51.90 (50.12)	0.43	0.59	0.61	51.27 (50.14)	0.46	0.80

### Validation on external dataset

The results of the experiment on an external validation data is displayed in Table 7 and shows the kernel function-‘edit distance normalized by the length of the longer sequence’ (${\mathbf {K}}{\mathbf {2}}$) achieved the best F1-score of **0.95** and **95%** accuracy. The ‘edit kernel’ (${\mathbf {K}}{\mathbf {1}}$) and ‘edit distance normalized by the number of common items’ (${\mathbf {K}}{\mathbf {3}}$) both achieved the same F1-score of **0.93** with **92.50%** and **93.75%** accuracy respectively. The ‘edit distance normalised by exponent of number of common items’ (${\mathbf {K}}{\mathbf {4}}$) achieved **0.90** F1-score and **0.95%** accuracy.

The results obtained from MKL applied to the validation dataset are displayed on Table 8. We combined the best performing kernels and obtained an F1-score of **0.97** and Accuracy of **96.75%**. In addition, we transformed the validation dataset into feature vectors with the Bag-of-Words representations and the results displayed in Tables 8 and 9. We obtained an F1 score of **0.95** with SVM implemented with the Laplace kernel on the Bag-of-Words features with accuracy of **96.52%**. The results of the deep learning experiments on the validation dataset are displayed in Table 10. It shows the LSTM model achieved an F1 score of **0.87%** and Accuracy of **85.05%**

**Table 7:** Best results obtained from classification with single kernels applied to the validation (peptide) data.

Kernel	F1	Acc (%)	Sen	Spec	nSv	%-Eig
$K_1$	0.90	90.58	0.98	0.85	0	156.35
$K_2$	0.87	85.71	0.79	0.97	0	156.35
$K_3$	0.93	92.86	0.93	0.93	0	156.35
$K_4$	0.92	90.56	0.91	0.93	0	156.35

**Table 8:** Best MKL results obtained by combining the top 7 kernels applied to the validation (peptide) data.

Kernels	F1	Acc (%)	Sen	Spec
Top 7 kernels	0.97	96.75	1.00	0.93

**Table 9:** Performance result obtained with logistic regression and SVM applied to Bag-of-Words and Binary Bag-of-Words feature representation of the validation (peptide) data.

Models		Bag-of-Words				Binary Bag-of-Words			
		F1	Acc (%) ± (std)	Sen	Spec	F1	Acc (%) ± (std)	Sen	Spec
SVM	Linear	0.92	92.01(27.11)	0.93	0.91	0.91	91.49 (28.00)	0.92	0.91
	Poly	0.93	93.56 (25.00)	0.93	0.94	0.92	92.27 (27.00)	0.94	0.91
	RBF	0.92	92.27 (27.00)	0.89	0.96	0.92	92.01 (27.00)	0.90	0.94
	Exp RBF	0.91	90.21 (30.00)	0.85	0.98	0.91	90.27 (30.00)	0.85	0.97
	Laplace	0.95	95.36 (21.00)	0.94	0.96	0.92	91.75 (28.00)	0.91	0.93
Logistic regression		0.92	92.01 (27.10)	0.92	0.92	0.92	92.53 (26.30)	0.92	0.93

**Table 10:** Performance result obtained with deep learning LSTM and MLP applied to Bag-of-Words and Binary Bag-of-Words feature representation of the validation (peptide) data.

Models		Bag-of-Words				Binary Bag-of-Words			
		F1	Acc (%)	Sen	Spec	F1	Acc (%)	Sen	Spec
Deep learning	MLP	0.54	60.76 (48.98)	0.53	0.67	0.46	59.49 (49.25)	0.89	0.63
	LSTM	0.83	84.54 (36.20)	0.80	0.87	0.87	85.05 (35.70)	0.79	0.95

## Discussion

This study set out to implement a machine learning predictive prognosis model capable of identifying healthy patients at risk of developing type 2 diabetes given an occurrence of elevated BP of 130/80 mmHg. Results show that the proposed EHR kernel framework implemented via edit-distance based kernels achieves a high predictive performance even when there exist significant disparities in EHR sequence size and sampling regularity, something that presents representational difficulties in standard machine learning approaches.

The initial round of experiments indicate that certain data modalities hold more intrinsic predictive value, with the Clinical dataset having the best single kernel performance; an indication that patient medical histories hold most informative value regarding patient behavior. However, extracting all the data into a composite ‘single view’ dataset outperformed the result achieved by the Clinical dataset alone; despite a similar F1-score of 0.74, the single view dataset achieves a higher sensitivity of 0.98. (Although its specificity of 0.51 means that it fails to identify half of those less likely to succumb to the disease, we nevertheless accept the outcome on the basis that the anticipated intervention of prescribing a healthy lifestyle to people at risk of the disease is not deemed harmful to healthy patients).

The findings show that we can apply MKL to combine poor base kernels to achieve a significantly better model in an EHR context. In particular, the we believe that this finding give strong support for kernel-based symbolic data representation as a suitable approach for modeling longitudinal clinical data. However, in most cases MKL results in a relatively high number of support vectors (in terms of which the final decision boundary is described). 80 % of datapoints are support vectors in the Recall, Refer, and Repeat datasets, while 71% and 74% of the data points are support vectors in the Clinical and single view datasets respectively. This may suggest a potential for overfitting if too many kernels are used; however, the MKL experiment combining 24 kernels in Table 4 in fact had a lower number of support vectors (41 which is 26% of the data points).

By contrast, the best F1-score of 0.63 achieved by the more conventionally representative BoW features was obtained via SVM model implemented with Laplace kernel on the binary BoW features and logistic regression applied to the Binary Bag-of-Words. Directly comparing with the results obtained via our proposed model applied to the same single view dataset, we see a significant improvement in the classification performance via the featureless edit-based kernel approach; in terms of the F1-score, accuracy and Sensitivity, both single and MKL models outperformed the BoW results. We argue that this superiority makes our proposed model the preferred choice for predictive modeling with disparate longitudinal EHR data.

The kernel alignment evaluation of the 4 kernel functions show the base ‘edit kernel’ and the ‘edit kernel normalized by the length of the longer sequence’ are in agreement on all datasets with scores closer to 1. However, we obtained the opposite with the ‘edit kernel normalized by the exponent of the number of common items’ kernel applied to the Clinical, Therapy and Test datasets. Kernel alignment scores approaching 0 indicate the degree of disagreement. All of our kernel functions on the other hand, appear in agreement on the Recall, Refer and Repeat datasets. Despite showing the least agreement with the target, ‘edit kernel normalized by the exponent of the number of common items’ with post kernel matrix spectral modification performed well on all datasets. The additional process to centralize the kernel matrices did not appear to affect the classification results. Findings also show that the alignment evaluation may not be suitable as a stand-alone metric for determining how useful a kernel function may be in terms of classification predictive performance.

In practice, it is difficult to establish a priori similarity measures that will yield the best classification result since such qualities are inherently data specific (in effect, the ‘no free lunch’ theorem). The edit distance computation is based on minimizing a weighted edit cost incurred in transforming one sequence into another. This however ignores any effects of the cost on the size of both sequences. We applied three methods of normalizing the edit cost computation to this effect and observed a varying degree of performance on the datasets. The performance (F1-score) of the kernel functions on the Recall, Refer and Therapy datasets are much closer. Normalization with the exponent of the number of common items performed best (F1-score) on the Clinical dataset. The variation of the normalising values generated with this kernel is more significant between pairs of sequences with similar items than with the other two normalising methods implemented. The pairs with a greater number of identical items are normalised by a large factor thereby making their similarity score smaller and thus indicating they are much closer than without normalization. The results of our experiment indicates that this had more effect on the separability of the data.

The success of deep learning in part relies on the availability of very large training datasets and computational resources. Deep learning constrained to a small sample-sized sparse dataset consisting of 158 patients, as in the current case, is generally not feasible (techniques such as data augmentation or transfer learning can be applied to overcome this problem to a certain extent: however, such measures are beyond the scope of this study). Nonetheless, the results obtained from experiments show that the kernel framework presents an alternative strategy for addressing classification tasks with uneven-length clinical sequences. Moreover, the computational efficiency of processing high dimensional features with small sample-sized examples, as a feature of the kernel framework, constitutes a salient advantage over deep learning.

The results obtained from experimenting with the validation set shows the kernel method had comparable, though less significant, performance results against those arising from the Bag-of-Words features. Applying MKL with the top 7 performing kernels achieved a comparable result to those of the single kernels; adding more kernels to the mixture degraded the performance (the same leave one out cross validation used on the primary dataset was adopted for the validation data experiment for comparative reasons). By validating our model on a dataset from a different domain, we are able to show the good classification performance obtained further underpins our hypothesis that the proposed framework can be applied to uneven-length and irregularly sampled EHR data.

As a further note, while LibSVM solver is in fact capable of handling non convex optimization problems, the proposed spectral modification to guarantee PSD kernels achieved a higher score than learning directly from indefinite kernels. Clipping and flipping the negative eigenvalues also performed better than shifting or squaring.

## Conclusion

In this study, we proposed the edit distance based kernel framework as a viable approach for overcoming the problems with symbolic EHR data, specifically in dealing with irregularly sampled uneven length longitudinal data. The case study findings show that the proposed framework has the potential to be implemented as a disease prognosis tool, providing a means to identify those at risk of developing type 2 diabetes from a prior incident of elevated blood pressure of 130/80 mmHg at the primary care level.

We propose that the outlined featureless edit kernel strategy may represent a generally preferable form of EHR based machine learning on the basis of its implicit retention of all clinically relevant information that may otherwise be lost in the feature representation process.

## Limitations

Given the scope of the investigation, namely to establish the inherent suitability of featureless methods for EHR on the basis of their retention of all symbolic and real-valued data on an equal footing, it is not within the experimental scope (or part of the argument) to eliminate the inverse-corollory that feature-based methods are inherently always information losing. Indeed there will invariably be many situations in which the intrinsic feature richness is such that this is not the case, and some overparameterized situations in which information loss (as opposed to noise loss) may be concretely useful.

## Supplementary Information

Supplementary Information.

## Data Availability

The datasets analysed during the current study are available on Github repository, [https://github.com/Nanomsky/KernelFrameworkPaper].
