# Supplementary material for: A novel kernel based approach to arbitrary length symbolic data with application to type 2 diabetes risk
Source: Sci Rep. 2022 Mar 23;12:4985. doi: 10.1038/s41598-022-08757-1 (PMC8943170; doi:10.1038/s41598-022-08757-1)
Supplement: Supplementary file 1 — Supplementary Information. [file 41598_2022_8757_MOESM1_ESM.pdf]

Supplement to "A Novel Kernel Based Approach To Arbitrary Length Symbolic Data With Application To Type 2 Diabetes Risk"

Nnanyelugo Nwegbu[1,*]**, Santosh Tirunagari**[2]**, and David Windridge**[+]

[1,2,+]School of Science and Technology, Department of Computer Science, Middlesex University, London, NW4 4BT, UK
[*]Correspondence and requests for materials should be addressed to N.N. (NN133@live.mdx.ac.uk)

ABSTRACT

Datasets

The data in its original form is a synthetic derivative of clinical data created for quality assurance purposes. It was previously used as clinical data for testing third-party integration with Vision 3 GP IT; it thus represents EHR data stored in a relational database (it is not publicly available in its original form).

Data Preprocessing/Cleansing

Records with missing values were ignored from the extract.

1. **Clinical Table:** This data table has the journal entries;
    - 43 events without a read code and read term were deleted
    - The date format was changed to yyyymmdd
    - Deleting outliers
        - Example 102 had an item ("stopped smoking") recorded 19290101 with a gap of 44 years to the next item recorded -19730101
        - Example 30 had an item ("stopped smoking") recorded 19300101 with a gap of 30 years to the next item recorded '19600101'
        - Example 136 had an item recorded 19010701 with a gap of 39 years to the next item recorded "19390101"
    - Dates with the year but missing either month and day were set to year 01 01
    - Read Code, Read Term, event dates were extracted
2. **Recall Table:** This data table holds the recalls made. It contains the read code, read term ordered by the event date. No further preprocessing was done. The read code, read term and event date were extracted.

3. **Test Table:** This data table holds the test results for the examples. It contains various tests and measurements. 52 unique tests entities with 30 containing numeric values. The Event date, Test read codes, read term, and any corresponding values are extracted. Null or missing records are excluded.

4. **Refer Table:** The Refer table holds information on the referrals sent. The referral read code, read term and event dates are extracted. Null or missing records are excluded.

5. **Repeat Table:** The Repeat table contains repeat medication orders made. The drug name, dosage, term, form, event date were extracted.

6. **Therapy Table:** This has the acute prescriptions made. Similar to the repeat table, the drug name, dosage, term, form, event date were extracted.

Supplementary Method

Learning Algorithm - Support Vector Machine (SVM)

The underlying support vector machine (SVM) model adopted here is Wolfe-dualized version of the the standard soft margin classifier obtained via Lagrange maximization of the margin with respect to a linear constraint on classification performance:

$$\min_{\omega,b,\xi} \quad \frac{1}{2}\omega^T\omega \;+\; C\sum_{i=1}^{l}\xi_i \tag{1}$$

subject to

$$y_i(\omega^T\phi(x_i) \;+\; b) \;\geq\; 1-\xi_i$$

$$\xi_i \;\geq\; 0,\; i \;=1,...,l$$

where $\phi(x_i)$ maps x_i into a higher-dimensional space and $C > 0$. The regularization parameter C is chosen by the user to control the trade-off between margin and classification error.

Multi Kernel Learning - MKL

Several methods have been proposed and used to combine base kernels. Figure 3 of the manuscript displays the MKL framework for applying kernel functions to disparate data entities such that they may be combined as kernel matrices with weight σ. For the purpose of the experiment carried out here, we use SimpleMKL[1]. Given m basis kernels $K_m(x,y),(m=1,.....,m)$, the goal is to optimize their combination in the context of the above SVM classifier.

$$K(x,y) = \sum_{m=1}^{M} d_m K_m(x,y) \tag{2}$$

subject to

$$d_m \geq \; 0 \text{ and } \sum_{m=1}^{M} d_m = 1$$

Where d_m denotes the weight of the kernels. In addition to solving an SVM classifier

$$f(x) = \sum_{i=1}^{l} \alpha^* K(x,x_i) \;+b^* \tag{3}$$

b^* and α^* are coefficients of the SVM to be learned. Therefore the goal of the MKL solver is to learn both SVM coefficients and combination weights simultaneously. This can be achieved by solving

$$\min \frac{1}{2} \left\| \sum_i \alpha^* K(.,x_i) \right\|^2 + C \sum_i \xi \tag{4}$$

subject to

$$y_i \sum_i \alpha_i K(x_i, x_j) + y_i b \geq 1 - \xi_i$$

$$\xi_i \geq 0$$

$$\sum_{m=1}^{M} d_m = 1,\ d_m \geq 0$$

Kernel Evaluation and selection

The methods described in this section were used to evaluate the appropriateness of the respective kernel functions. These were applied to establish, rank and assess the quality of kernels.

Kernel Alignment

Kernel alignment[2], which is able to quantify the similarity between two kernel functions or between a kernel and a target, was here used to evaluate the quality of kernels developed. The kernel alignment of kernel K_1 and kernel K_2 is given as

$$A(K_1, K_2) = \frac{\langle K_1, K_2 \rangle}{\sqrt{\langle K_1, K_1 \rangle \langle K_2, K_2 \rangle}}$$

This can be viewed as the cosine of the angle between two bi-dimensional vectors K_1 and K_2.

For a given vector y of labels $\{+1, -1\}$, if we consider $K_2 = yy'$, the kernel target alignment is given as

$$A(K_1, yy') = \frac{\langle K_1, yy' \rangle}{\sqrt{\langle K_1, K_1 \rangle \langle yy', yy' \rangle}}$$

This is the (normalized) Frobenius inner product between the kernel matrix and the covariance matrix of the target vector, capturing the degree of agreement between a kernel and a given learning task[2].

Supplementary Result

Tables	K_1	K_2	K_3	K_4
Clinical	0.2259	0.2261	0.2261	0.3219
Recall	0.2177	0.2322	0.2390	0.3110
Refer	0.1831	0.1988	0.2030	0.4151
Repeat	0.1562	0.1591	0.1599	0.5248
Test	0.2500	0.2500	0.2500	0.2500
Therapy	0.0000	0.0000	0.0000	1.0000
All data	0.0000	0.0000	0.0000	1.0000

Table 1. Weights (sigma) obtained from multi kernel learning process

Table	K_1	K_2	K_3	K_4
Clinical	0.9735	0.9577	0.8862	0.6356
Recall	0.9212	0.9157	0.9065	0.8917
Refer	0.9833	0.9812	0.9797	0.9579
Repeat	0.9825	0.9728	0.9721	0.9124
Test	0.8383	0.8119	0.7607	0.5562
Therapy	0.9929	0.9926	0.9208	0.5379
All data	0.9577	0.9169	0.8219	0.2378

Table 2. Kernel Target Alignment scores obtained with the 4 kernel matrices derived from the data tables. This shows the alignment with the target labels

Deep Learning Architecture

Long Short Term Memory (LSTM)

The graph depicting the LSTM network applied to the Bag-of-words features are illustrated in the image below in Fig 1.

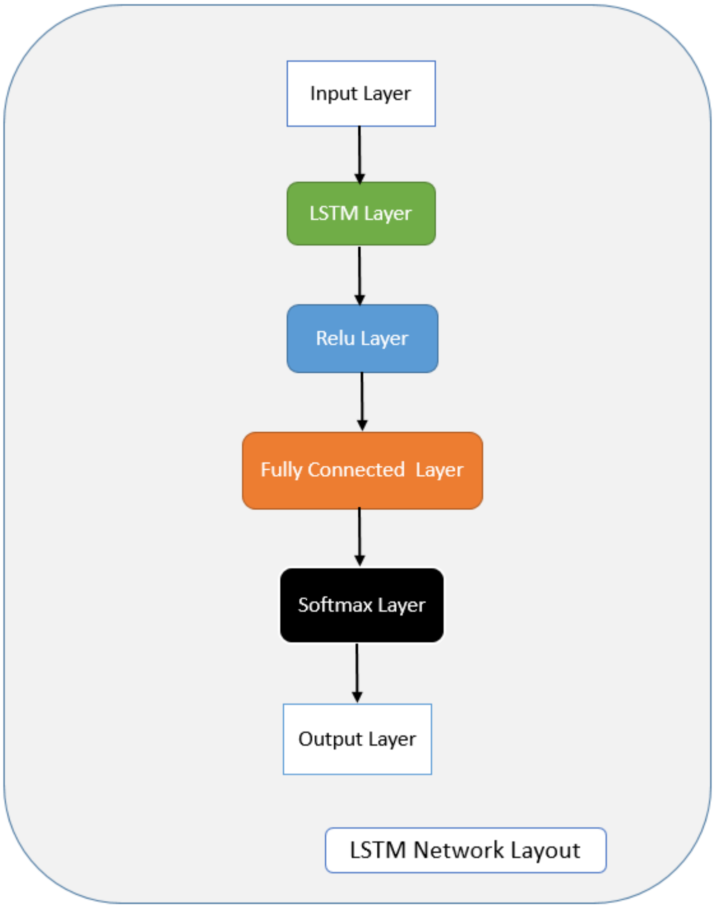

Figure 1. LSTM network design

The LSTM Matlab trained network parameters for the Bag-of-words features of the primary and validation data are uploaded into Github at the specified location below

[https://github.com/Nanomsky/KernelFrameworkPaper/tree/main/LSTM_Network])

- LSTM_EHR_BIN.mat
- LSTM_EHR_BOW.mat
- LSTM_Peptide_BIN.mat
- LSTM_Peptide_BOW.mat

Multi-Layer Perceptron (MLP)

The graph depicting the Multi Layer Perceptron architecture applied to the Bag-of-words features are illustrated in the image in Fig 2.

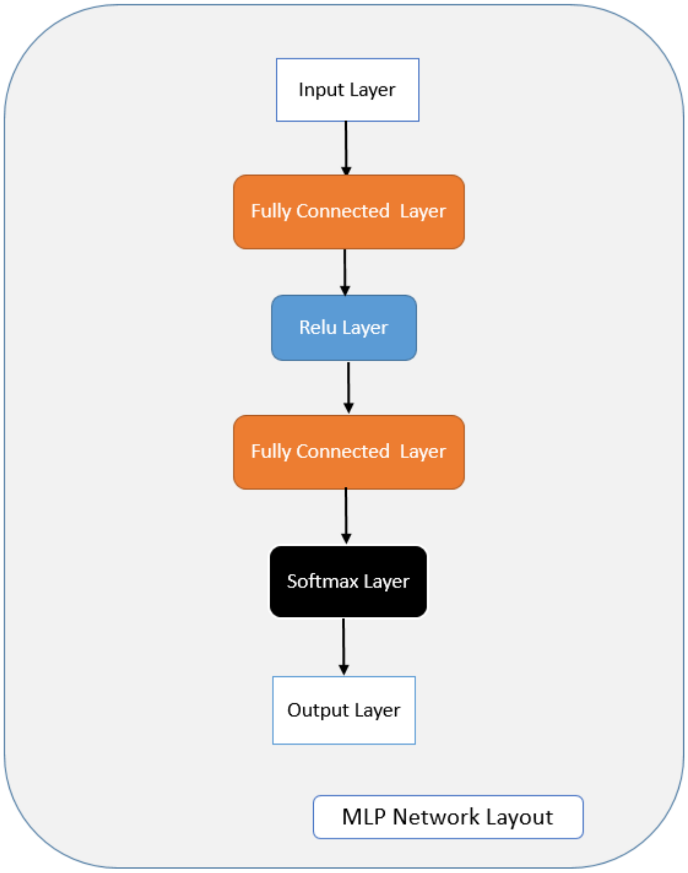

Figure 2. MLP network design

The MLP Matlab trained network parameters for the Bag-of-words features of the primary and validation data are uploaded into Github at the specified location below
[https://github.com/Nanomsky/KernelFrameworkPaper/tree/main/MLP_Network])

- MLP_EHR_BIN.mat
- MLP_EHR_BOW.mat

- MLP_Peptide_BIN.mat
- MLP_Peptide_BOW.mat

The files in both locations contain the details of the network layers, options, and hyper parameter weights for the Bag-of-words (binary) features with results displayed in Table 6 of the main text.

References

1. Rakotomamonjy, A., Bach, F. R., Canu, S. & Grandvalet, Y. SimpleMKL. *J. Mach. Learn. Res.* **9**, 2491–2521 (2008).
2. Cristianini, N., Kandola, J., Elisseeff, A. & Shawe-Taylor, J. On kernel-target alignment. *Adv. Neural Inf. Process. Syst. 14* 367—-373, DOI: 10.1.1.23.6757 (2002).
